# Atrophy signaling pathways in respiratory and limb muscles of guinea pigs exposed to chronic cigarette smoke: role of soluble guanylate cyclase stimulation

**DOI:** 10.1152/ajplung.00258.2022

**Published:** 2023-03-07

**Authors:** Víctor Ivo Peinado, Maria Guitart, Isabel Blanco, Olga Tura-Ceide, Tanja Paul, Joan Albert Barberà, Esther Barreiro

**Affiliations:** ^1^Department of Pulmonary Medicine, Hospital Clínic, Institut d’Investigacions Biomèdiques August Pi i Sunyer (IDIBAPS), University of Barcelona, Barcelona, Spain; ^2^Department of Experimental Pathology, Institut d’Investigacions Biomèdiques de Barcelona (IIBB), CSIC-IDIBAPS, Barcelona, Spain; ^3^Centro de Investigación en Red de Enfermedades Respiratorias (CIBERES), Instituto de Salud Carlos III, Madrid, Spain; ^4^Respiratory Medicine Department, Muscle & Lung Cancer Research group, IMIM-Hospital del Mar, Parc de Salut Mar, MELIS, UPF, PRBB, Barcelona, Spain; ^5^Pneumology Department, Girona Biomedical Research Institute (IDIBGI), Girona, Spain; ^6^Faculty of Medicine, Institute of Pathology, Ludwig-Maximilians University Munich, Munich, Germany

**Keywords:** cGMP, COPD, muscle atrophy, pharmacology

## Abstract

Skeletal muscle dysfunction in chronic obstructive pulmonary disease (COPD) is characterized by a significant reduction in muscle strength and endurance. Preclinical studies show that stimulation of the soluble guanylate cyclase (sGC)-cGMP pathway attenuates muscle mass loss and prevents cigarette smoke-induced oxidative stress, indicating that pharmacological activation of the guanylyl cyclase pathway in COPD may provide a beneficial therapeutic strategy that reaches beyond the lung. In this study, conducted in an animal model of COPD, we first set out to assess the effect of cigarette smoke (CS) on biomarkers of muscle fatigue, such as protein degradation and its transcriptional regulation, in two types of muscles with different energy demands, i.e., the diaphragm and the gastrocnemius muscle of the limbs. Second, we evaluated the administration of an sGC stimulator on these markers to study the potential efficacy of such treatment in the recovery of skeletal muscle function. Exposure to CS led to weight loss, which was associated in the gastrocnemius with increased levels of proteolytic markers of muscle atrophy (MURF-1, Atrogin-1, proteasome C8 subunit 20 s, and total protein ubiquitination), whereas the size of fast-twitch muscle fibers decreased significantly. Long-term treatment with the sGC stimulator BAY 41-2272 resulted in a significant reduction in gastrocnemius levels of the aforementioned proteolytic markers, concomitant with a weight recovery and increased cGMP levels. Remarkably, levels of some of the analyzed biomarkers differed between respiratory and limb muscles. In conclusion, targeting sGC might exert beneficial effects on muscle alterations in patients with COPD.

## Graphical abstract

**Figure:**
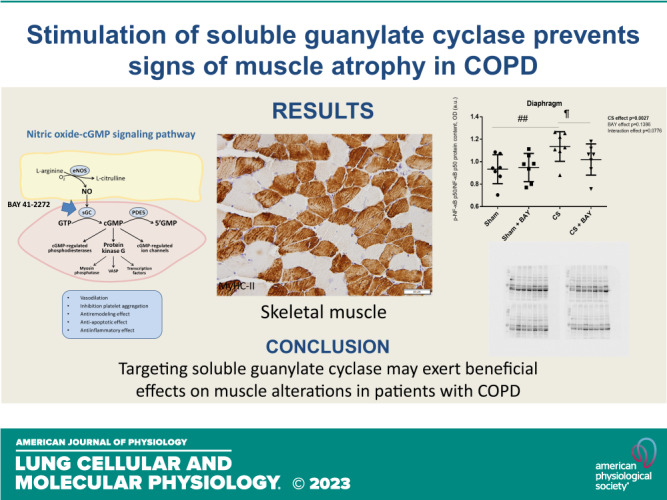

## INTRODUCTION

Skeletal muscle dysfunction in chronic obstructive pulmonary disease (COPD) is characterized by a significant reduction in muscle strength and endurance (1). Its etiology, closely related to smoking, is multifactorial, but systemic inflammation and local and systemic oxidative stress are the factors that contribute most to impaired muscle performance (2). Although the precise mechanisms by which cigarette smoke influences skeletal muscle loss and atrophy are not fully elucidated, on the basis of increased oxidant production, evidence suggests that transient and repeated episodes of redox imbalance induced by cigarette smoke oxidize key proteins involved in either the metabolism or the function of muscle fibers (3, 4). One of the key muscle proteins that is susceptible to oxidation is soluble guanylate cyclase (sGC), which is involved in the regulation of multiple functions; its expression is reduced in lung biopsies from smokers and in patients with COPD, correlated with disease severity (5–7). The deleterious effects of acute, subacute, and chronic cigarette smoke (CS) exposure on the expression of sGC have been demonstrated in different animal models, at both the mRNA and protein levels (7, 8).

In the skeletal muscle, sGC is the primary cyclic GMP (cGMP) source and its expression has been demonstrated to be greater in metabolically oxidative muscles, suggesting greater cGMP synthesis capacity along with muscle-specific phenotypic differences in sGC function (9, 10). Moreover, sGC activity exhibits partial nNOS dependence (11, 12). In partially sGC-deficient animal models, muscles exhibited reduced fatigue resistance. Furthermore, changes in the redox state of the heme iron moiety within the β1 subunit or the cysteine residues within the sGC protein has been shown to greatly modify the sensitivity and efficiency of the sGC protein in catalyzing the production of cGMP (13). Therefore, redox variations can affect muscle physiology and a wide range of functions.

Increased ROS levels due to CS exposure can also activate the proteasome and lead to increased protein degradation (14). Indeed, it is known that the skeletal muscle wasting process that accompanies COPD is due in part to skeletal muscle protein degradation via the ubiquitin-proteasome system (UPS) (15). In cultured skeletal muscle cells, exposure to reactive oxygen species (ROS) triggers significant increases in proteasomal activity and enhanced expressions of the E3 ligases MURF1 and Atrogin-1 (16). Evidence that supports increased UPS activity in limb and ventilatory muscles of patients with COPD is largely based on measuring 20S proteasomal activity and documenting the expression levels of MURF1 and Atrogin-1 (17, 18).

Therapeutic strategies based on stimulation of the sGC-cGMP pathway in animal models of COPD have shown, in addition to attenuating the loss of muscle mass, reductions in cigarette smoke-induced oxidative stress (7, 19). A network analysis of differentially expressed genes in the lung in these animal models showed that CS exposure induces serine-threonine kinases and some phosphatases that regulate the phosphorylation of proteins from the MAPK pathway (20). Moreover, the same study revealed that CS also activates a set of proteases and peptidases associated with lung autophagy. Finally, it is interesting to note that, among the biological processes activated in the lung, were some related to skeletal muscle cell differentiation. Importantly, oral administration of an sGC stimulator decreased and normalized the expression of kinases and proteases, suggesting the potential therapeutic effect reversing protein degradation.

Accordingly, on the basis that alterations to the sGC-cGMP-PKG axis may underlie skeletal muscle atrophy in COPD, we hypothesized that pharmacological activation of the guanylyl cyclase pathway may provide a beneficial therapeutic strategy in the treatment of muscle dysfunction in COPD. In this study, focused on skeletal muscle toxicity induced by CS exposure in guinea pigs, first, we aimed to evaluate the effect of CS on biomarkers of muscle fatigue, such as protein degradation and its transcriptional regulation, in two muscle types with different loads and energetic demands, i.e., the diaphragm and the limb gastrocnemius muscles. Second, we evaluated the therapeutic administration of a sGC stimulator on these markers to study the potential efficacy of such treatment in the recovery of skeletal muscle function.

## MATERIALS AND METHODS

### Animal Experiments

Twenty-eight male Dunkin-Hartley guinea pigs (Harlan Laboratories, Inc., Indianapolis) with ages between 4 and 5 wk were provided with standard guinea pig chow and water supplemented with ascorbic acid (Bayer Hispania, Sant Joan Despí, Spain) ad libitum. Animals were housed in nonmetabolic cages under controlled conditions.

### Exposure to CS

After 2 wk of adaptation, animals were randomly divided into four groups: *1*) a sham-exposed control group that received the control vehicle (polyethylene glycol 400, 5 mL/kg; Fluka Analytical, Sigma-Aldrich, Steinheim, Germany) (*n* = 7); *2*) a sham-exposed treatment group that received the sGC stimulator BAY 41–2272 (Bayer AG, Leverkusen, Germany) (*n* = 7); *3*) a CS-exposed control group that received the vehicle only (*n* = 7); and *4*) a CS-exposed treatment group that received BAY 41–2272 (*n* = 7). CS exposure was conducted as previously described (20–22), with guinea pigs exposed to the smoke of 6 cigarettes (3R4F, Kentucky University Research, Lexington, KY) per day, 5 days a week, for 3 mo. After CS exposure, animals received daily doses of either freshly prepared BAY 41–2272 in suspension at a dose of 3 mg/kg by oral gavage or an equivalent amount of the vehicle. The condition of the guinea pigs was assessed daily.

### In Vivo Measurements in the Guinea Pigs

In all study animals, body weight was measured once a week. Body mass index (BMI) was calculated by dividing the bodyweight by the square of the body length.

### Euthanasia and Sample Collection

Animals were euthanized by an overdose of phenobarbital (200 mg/kg ip; Sigma-Aldrich, Steinheim, Germany). Immediately, the gastrocnemius and diaphragm muscles were dissected and obtained in full for the purpose of the study. Muscle samples were snap-frozen in liquid nitrogen and stored frozen at −80°C to be further used for the molecular analyses or paraffin-embedded to be used for the assessment of muscle structural abnormalities and fiber type composition and morphometry.

### Ethical Statement

All procedures were approved by the ethics committee for animal experimentation of the University of Barcelona (registry: 383/15). An initial set of assessments in these animals has been previously published (20).

### Biological Analyses

#### cGMP assay in muscle tissue.

Quantitative measurement of cGMP was assessed by in vitro competitive ELISA using a commercial kit (cGMP ELISA Kit, ab133052, Abcam, Cambridge, CB2 0AX, UK) according to the manufacturer’s instructions. Briefly, around 50 mg of frozen tissue was ground to powder in a stainless steel mortar immersed in liquid nitrogen. Then, the powdered tissue was weighed and 10 volumes of cold 5% trichloroacetic acid (TCA) was added, vortexed, and centrifuged at 1,500 *g* for 10 min at 4°C. The supernatant was collected and 3 volumes of ether saturated in water were added, shaken and the ether phase discarded allowing the samples to evaporate to a volume of 50 µL. Finally, they were reconstituted with 250 µL of buffer. The concentration of cGMP was measured in duplicate in an ELISA reader at 405 nm against a standard curve. In parallel, the protein concentration was determined in the same sample by using the Bradford method (Protein Assay Dye Reagent Concentrate, Bio-Rad) and the concentration in the sample was expressed per mg of protein.

#### Muscle fiber counts and morphometry.

Three-micrometer muscle paraffin-embedded sections from gastrocnemius and diaphragm muscles of all study groups were rehydrated and incubated for 40 min in 1 mM EDTA (with 0.05% Tween, pH 8) at 95°C. Then, endogenous peroxidases were blocked with 6% H_2_O_2_ for 15 min before slides were incubated for 1 h with the primary antibodies anti-MyHC-I (anti-myosin skeletal slow, Merck Life Science, Darmstadt, Germany) or anti-MyHC-II (anti-myosin skeletal fast, Merck Life Science) at room temperature. Slides without primary antibody were used as negative controls. Slides were washed and incubated for 30 min with horseradish-conjugated universal secondary antibody (Polystain 1-step Kit, HRP for DAB, mouse and rabbit, no chromogen, Neo Biotech, Nanterre, France), followed by diaminobenzidine [DAB concentrated (20×) Kit for 2400 slides; 12 mL, Neo Biotech] for 5 min as a substrate. Nuclei were counterstained with hematoxylin for 2 min followed by the dehydration and mounting of tissue sections in DPX. Myofibers positively stained with the anti-MyHC type II antibody are stained in brown and type I fibers were not stained (white color). The cross-sectional area and proportions of type I and type II fibers were assessed using a light microscope (Olympus, Series BX50F3, Olympus Optical Co., Hamburg, Germany) coupled with an image-digitizing camera (Pixera Studio, version 1.0.4, Pixera Corporation, Los Gatos, CA) and a morphometry program (Image J, National Institutes of Health). At least 100 fibers were measured and counted in each muscle specimen from all groups of guinea pigs (23).

#### Muscle structure abnormalities.

To quantify muscle injury at a microscopic level we followed the methodology proposed by Macgowan et al. (24) for the determination of the normal and abnormal fractions. Briefly, three-micrometer paraffin-embedded sections from the muscle specimens of the three study groups were used to assess the proportions of muscle abnormalities (23). Samples were stained with hematoxylin-eosin. Images were captured at a magnification of ×400 under the light microscope (Olympus BX 61, Olympus Corporation) using an image-digitizing camera (Olympus DP 71, Olympus Corporation). A grid of 63 point-intercepts (7 × 9 rectangular pattern) was superimposed onto the image of the muscle cross section. Each point-intercept was assigned to a specific category and entered into the software. Categories for point counting were defined as follows: *1*) normal muscle, i.e., polygonal fiber, acidophilic cytoplasm, plasma membrane, peripheral muscle nuclei; *2*) internal nucleus, i.e., fiber with at least one muscle nucleus located internally; *3*) inflammatory cell, i.e., round-shaped nucleus in interstitial space; *4*) lipofuscin, i.e., fiber containing deposits of yellow-brown pigmentation at least size of a muscle nucleus; *5*) abnormal viable, i.e., small fiber with two or more oblique angles or with basophilic peripheral sarcoplasm; *6*) inflamed/necrotic, i.e., fiber containing inflammatory cell(s), necrotic mass of inflammatory cells, and muscle debris; *7*) vessel; and *0*) no count. The area fraction for each category was defined as the percentage of points that fell on each of these traits relative to the total number of points superimposed on all viable fields (all features except for categories *0* and *7*) of each cross section. The area fraction of normal muscle was equivalent to the proportions of points falling in *category 1*, whereas the area fraction of abnormal muscle was determined by calculation of the proportion of points included in the other categories (*categories 2 to 6*).

#### Terminal deoxynucleotidyl transferase-mediated dUTP nick-end labeling assay.

In three-micrometer paraffin-embedded sections of muscle specimens, apoptotic nuclei were determined using the TUNEL assay (ApopTag Peroxidase In Situ Apoptosis Detection Kit, Merck Millipore, Burlington). The manufacturer’s instructions and previously published studies were followed (25). In brief, during apoptosis fragments of genomic DNA can be generated. These strand breaks in the DNA sequence can be identified by labeling 3′-OH terminal groups with modified nucleotides in an enzymatic reaction catalyzed by the terminal deoxynucleotidyl transferase (TdT) enzyme. Muscle sections were fixed, permeabilized and immediately incubated with the TUNEL Working Strength TdT Enzyme and the anti-Digoxigenin Conjugate. TdT catalyzed the adding of digoxigenin-dNTP at 3′-OH terminal groups in single- and double-stranded DNA. After washing, the sections were incubated with an anti-digoxigenin antibody conjugated with peroxidase, which resulted in brown color on reaction. Negative control experiments, in which TdT enzyme was not added, were also performed. Apoptotic nuclei appeared brown in color, whereas negative nuclei were green (methyl green counterstaining). Only nuclei located within the muscle fiber boundary were counted in the study. Positive nuclei and the total number of nuclei were counted by two trained observers (correlation coefficient 95%). Apoptotic nuclei were expressed as the percentage of the TUNEL-positive nuclei to the total number of counted nuclei (25). A minimum of 300 nuclei were counted in each muscle specimen for all the study groups.

#### Immunoblotting.

Protein levels of proteolytic (MURF1, Atrogin-1, 20 s proteasome subunit C8 and total ubiquitinated proteins), apoptotic (Bax, Bcl-2, and caspase-3), and autophagy (p62 and LC3B) markers, as well as total protein levels and phosphorylated ratios of nuclear factor κB (NF-κB) pathway markers (NF-κB p50 subunit, p-NF-κB p50/NF-κB p50, NF-κB p65 subunit, and p-NF-κB p65/NF-κB p65), MAPK pathway markers (p38 MAPK and p-p38 MAPK/p38 MAPK), and Forkhead box O (FOXO) pathway markers (FOXO3 and p-FOXO3/FOXO3) were analyzed using immunoblotting procedures, as previously described (26–28). Briefly, frozen samples from all experimental groups were homogenized in lysis buffer: 50 mM HEPES, 150 mM NaCl, 100 mM NaF, 10 mM Na pyrophosphate, 5 mM EDTA, 10% glycerol, 0.5% Triton-X, 5 µg/mL aprotinin, 2 µg/mL leupeptin, 100 µg/mL PMSF and 10 µg/mL pepstatin A. Afterward, samples were centrifuged at 3,600 rpm for 30 min and protein levels were determined in the supernatant using the Bradford method (Bio-Rad). Thirty micrograms of total protein were loaded on gels, then proteins were separated by electrophoresis, transferred to polyvinylidene difluoride (PVDF) membranes, blocked with bovine serum albumin (BSA), and incubated overnight with the corresponding primary antibodies: MURF1 (anti-MURF1, Santa Cruz Biotechnology, Dallas), Atrogin-1 (anti- MAFbx, Santa Cruz Biotechnology), 20 s proteasome subunit C8 (anti-C8 antibody, Biomol, Plymouth Meeting, PA), total ubiquitinated proteins (anti-ubiquitin, Santa Cruz Biotechnology), Bax (anti-Bax, Santa Cruz Biotechnology), Bcl-2 (anti-Bcl-2, Santa Cruz Biotechnology), caspase-3 (anti-caspase-3, Santa Cruz Biotechnology), p62 (anti-p62/SQSTM1, Merck KGaA, Darmstadt, Germany), LC3B (anti-LC3B, Cell signaling technology, Danvers, MA), NF-κB p50 (anti- NF-κB p50, Santa Cruz Biotechnology), p-NF-κB p50 (anti- p-NF-κB p50, Santa Cruz Biotechnology), NF-κB p65 (anti- NF-κB p65, Santa Cruz Biotechnology), p-NF-κB p65 (anti-p-NF-κB p65, Santa Cruz Biotechnology), p38 (anti-p38 α/β, Santa Cruz Biotechnology), p-p38 [anti-p-p38 (Tyr 182) Santa Cruz Biotechnology], FOXO3 (anti-FoxO3, Origene Technologies, Rockville, MD), p-FOXO3 (anti-p-FoxO3, Origene Technologies), and the endogenous control glyceraldehyde-3-phosphate dehydrogenase (GAPDH) (anti-GAPDH antibody, Santa Cruz Biotechnology).

Antigens from all samples were detected with horseradish peroxidase (HRP)-conjugated secondary antibodies and a chemiluminescence kit (Pierce ECL Western Blotting Substrate, ThermoFisher Scientific, Waltham, MA). For each of the antigens, samples from the different groups were always detected in the same picture under identical exposure times. PVDF membranes were scanned with the Alliance Q9 Advanced Chemiluminescence Imager (UVITEC, Cambridge, UK). The optical densities of specific proteins were quantified using the software Alliance Q9 (UVITEC). The mean values of the optical densities obtained in each specific group were calculated. To validate equal protein loading among various lanes, the glycolytic enzyme GAPDH was used as the protein loading controls in all the immunoblots.

#### Oxidative stress markers in blood.

Plasma levels of 8-hydroxydeoxyguanosine (8-OHdG) were assessed by enzyme-linked immunosorbent assays (OxiSelect oxidative DNA Damage ELISA kit, Cell Biolabs, Inc. San Diego, CA) following the manufacturer’s instructions and previously reported methodologies (19).

### Statistical Analysis

**Figure 1.**
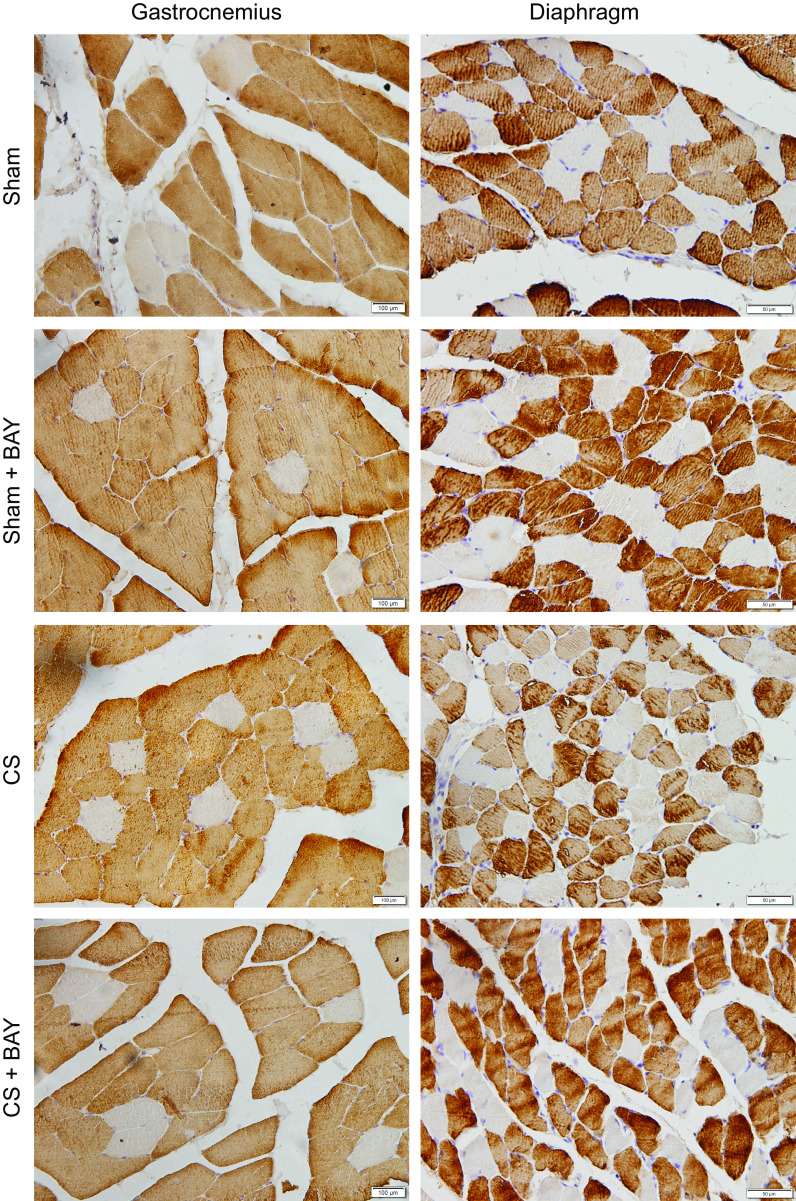
Representative immunostaining of MyHC-II in diaphragm and limb muscle tissue sections from one animal from each of the different experimental groups. Fast-twitch fibers are identified in brown (positive labeling), whereas slow-twitch fibers are shown in white (negative labeling). CS, cigarette smoke.

**Figure 2.**
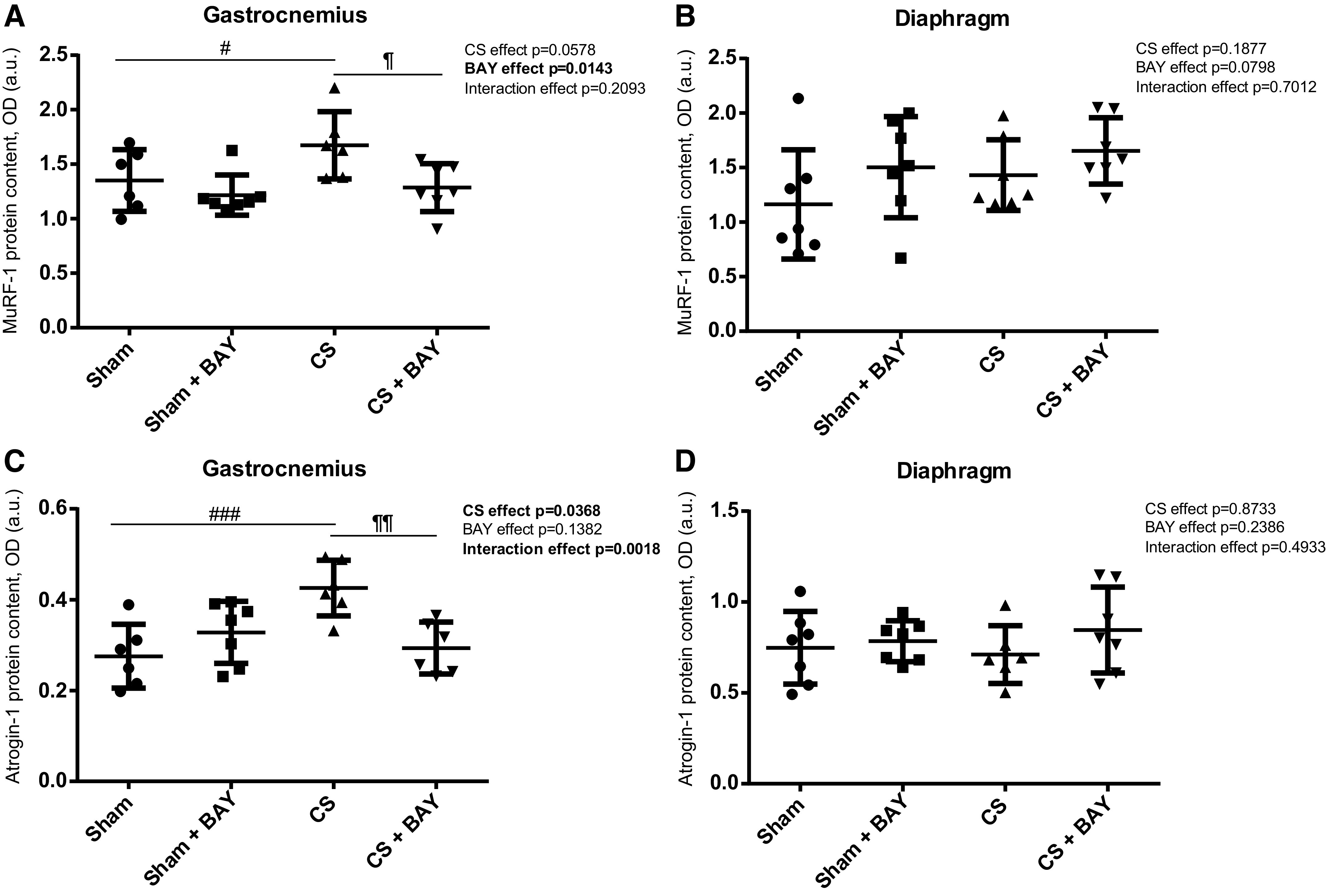
Mean values and standard deviation of MURF-1 protein content in the gastrocnemius (*A*), MURF-1 protein content in the diaphragm (*B*), atrogin-1 protein content in the gastrocnemius (*C*), and atrogin-1 protein content in the diaphragm (*D*) measured as optical density (OD) using arbitrary units (a.u.) in the muscles of the different guinea pig study groups. Potential differences between two groups were analyzed using contrast of marginal linear predictions. Statistical significance: #*P* ≤ 0.05, ###*P* ≤ 0.001 between the CS group and the sham control group; ¶*P* ≤ 0.05, ¶¶*P* ≤ 0.01 between the CS + BAY group and the CS group. The effect of CS and treatment and interaction effects are also indicated as actual *P* values for each variable. Bay, Bay 41–2272; CS, cigarette smoke.

**Figure 3.**
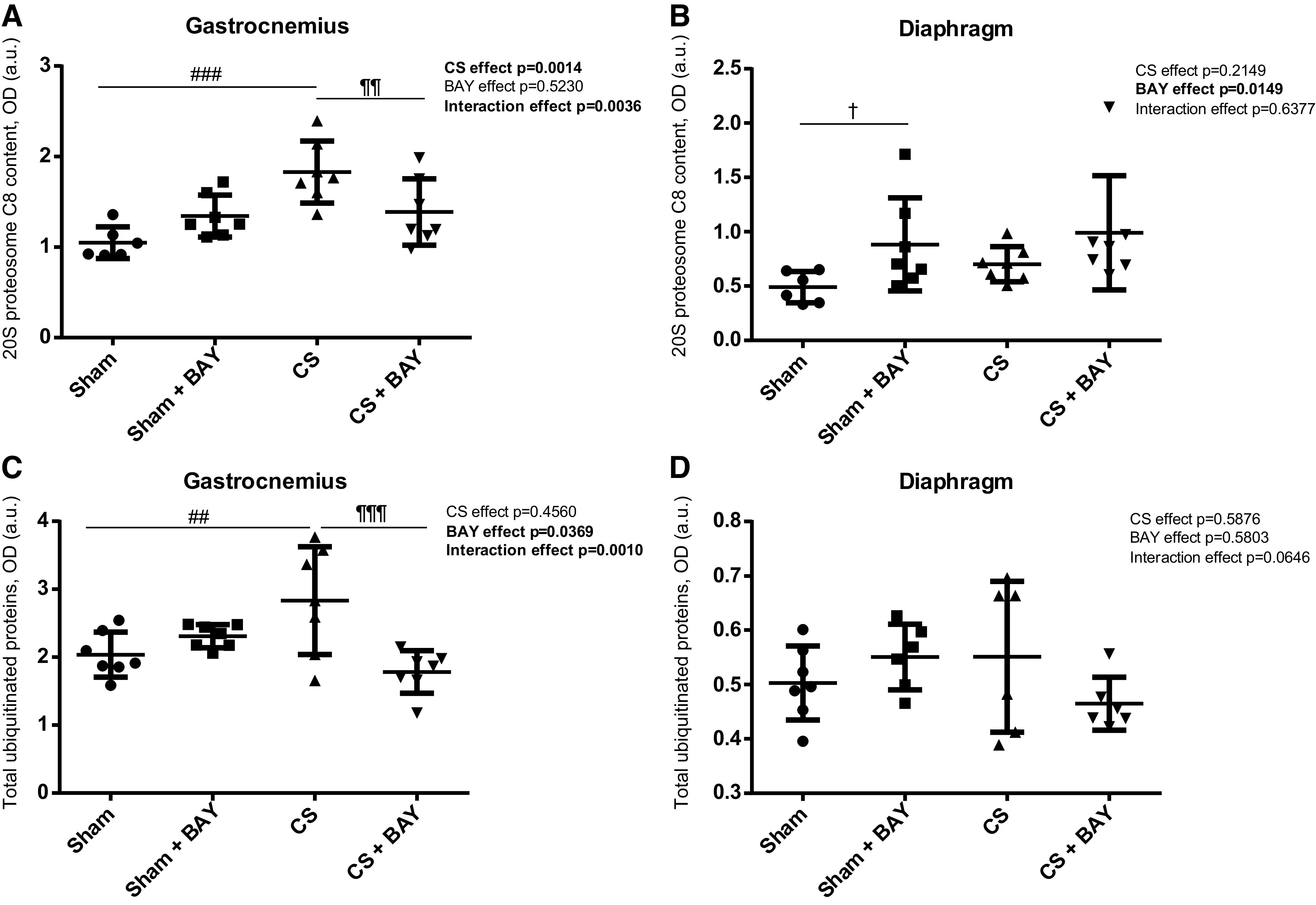
Mean values and standard deviation of the 20 s proteasome subunit C8 protein content in the gastrocnemius (*A*), 20 s proteasome subunit C8 protein content in the diaphragm (*B*), total ubiquitinated protein content in the gastrocnemius (*C*), and total ubiquitinated protein content in the diaphragm (*D*) measured as optical density (OD) using arbitrary units (a.u.) in the muscles of the different guinea pig study groups. Potential differences between two groups were analyzed using contrast of marginal linear predictions. Statistical significance: ##*P* ≤ 0.01, ###*P* ≤ 0.001 between the CS group and the sham control group; ¶¶*P* ≤ 0.01, ¶¶¶*P* ≤ 0.001 between the CS + BAY group and the CS group; †*P* ≤ 0.05 between the sham + BAY group and the sham control group. The effect of CS and treatment and interaction effects are also indicated as actual *P* values for each variable. Bay, Bay 41–2272; CS, cigarette smoke.

**Figure 4.**
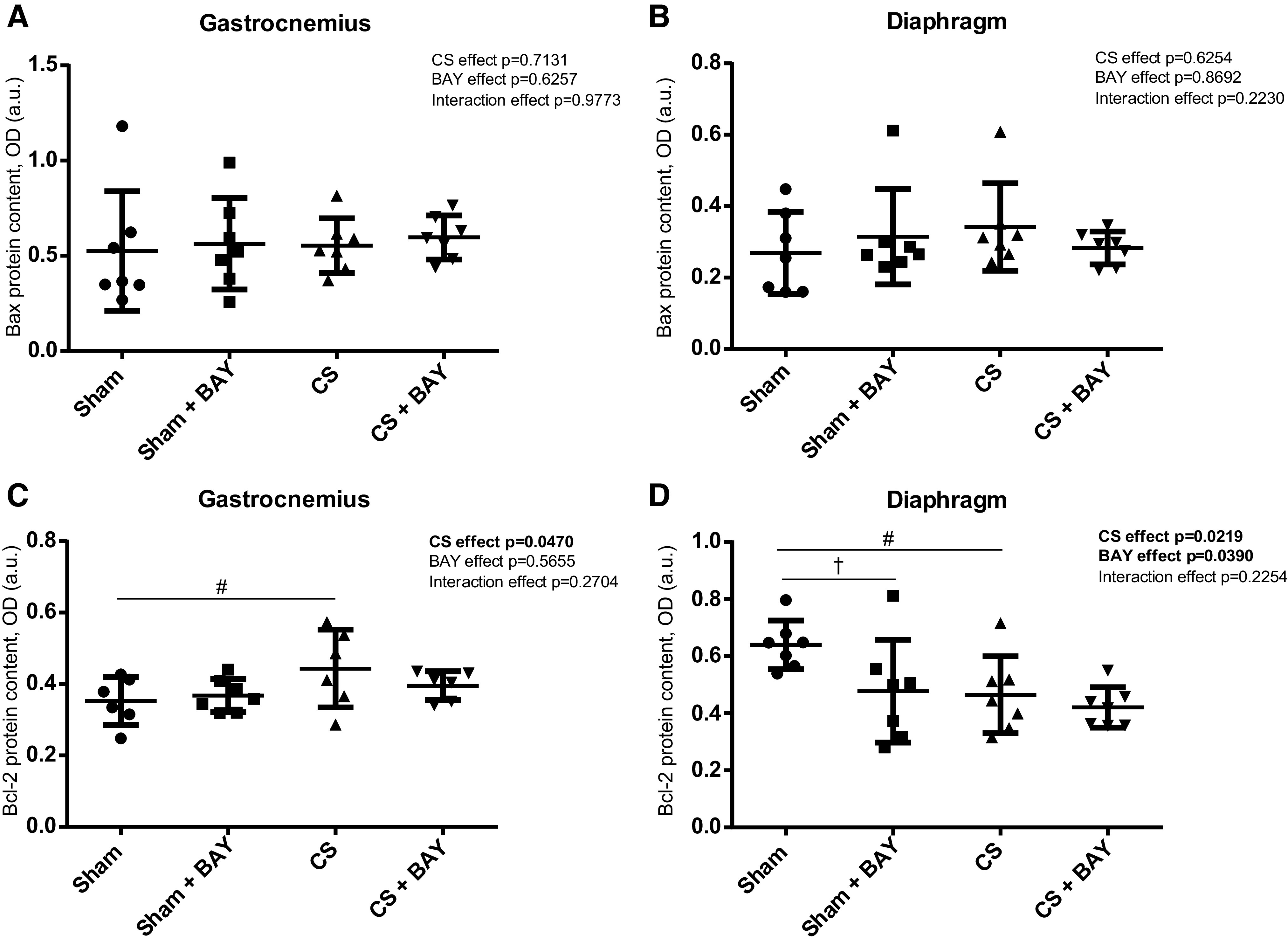
Mean values and standard deviation of the BAX protein content in the gastrocnemius (*A*), BAX protein content in the diaphragm (*B*), BCL-2 protein content in the gastrocnemius (*C*), and BCL-2 protein content in the diaphragm (*D*) measured as optical density (OD) using arbitrary units (a.u.) in the muscles of the different guinea pig study groups. Potential differences between two groups were analyzed using contrast of marginal linear predictions. Statistical significance: #*P* ≤ 0.05 between the CS group and the sham control group; †*P* ≤ 0.05 between the sham + BAY group and the sham control group. The effect of CS and treatment and interaction effects are also indicated as actual *P* values for each variable. Bay, Bay 41–2272; CS, cigarette smoke.

**Figure 5.**
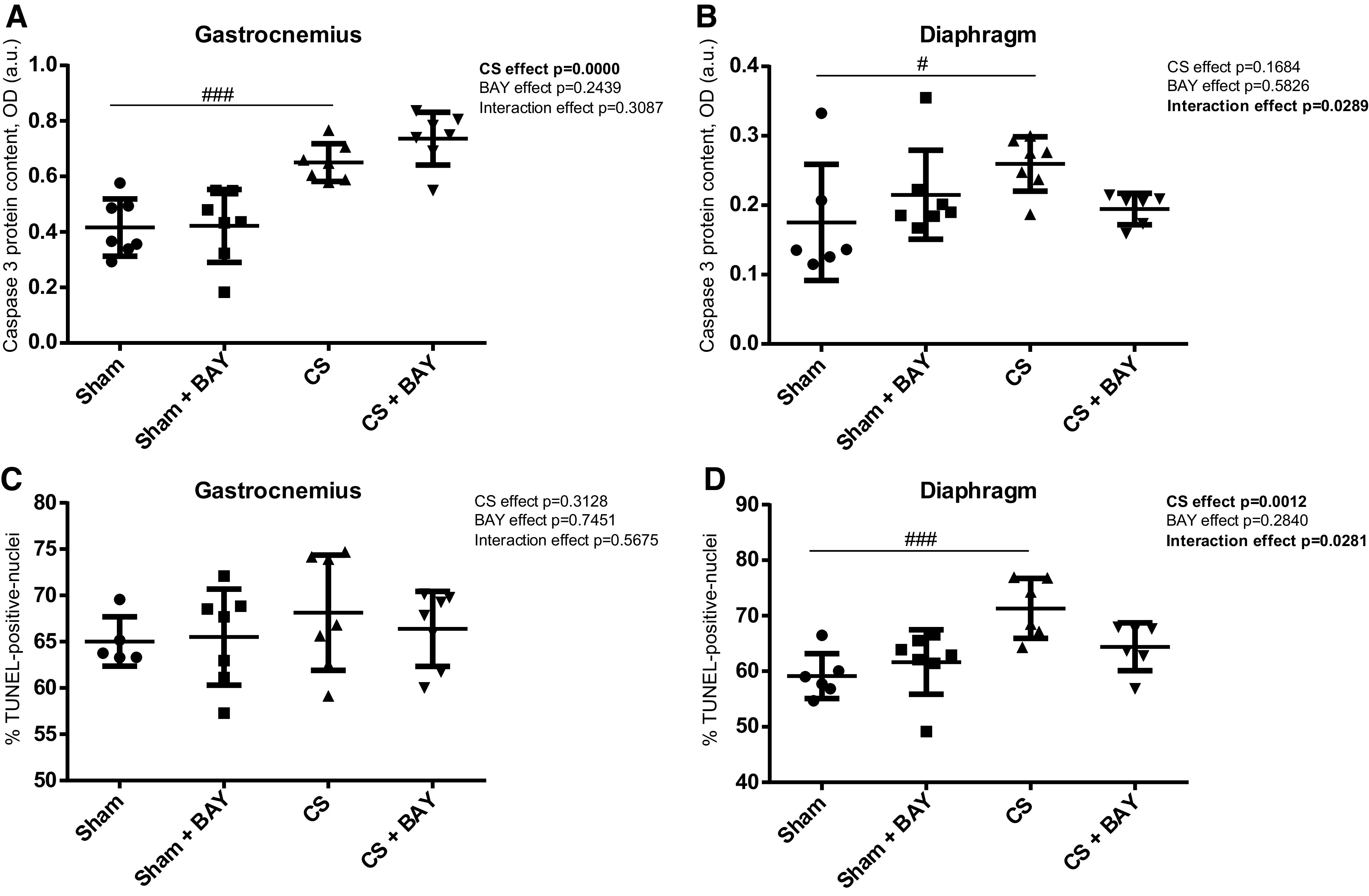
Mean values and standard deviation of the caspase-3 protein content in the gastrocnemius (*A*) and caspase-3 protein content in the diaphragm (*B*) measured as optical density (OD) using arbitrary units (a.u.) and percentage of TUNEL-positive nuclei in the gastrocnemius (*C*), and percentage of TUNEL-positive nuclei in the diaphragm (*D*) in the muscles of the different guinea pig study groups. Potential differences between two groups were analyzed using contrast of marginal linear predictions. Statistical significance: #*P* ≤ 0.05, ###*P* ≤ 0.001 between the CS group and the sham control group. The effect of CS and treatment and interaction effects are also indicated as actual *P* values for each variable. CS, cigarette smoke.

**Figure 6.**
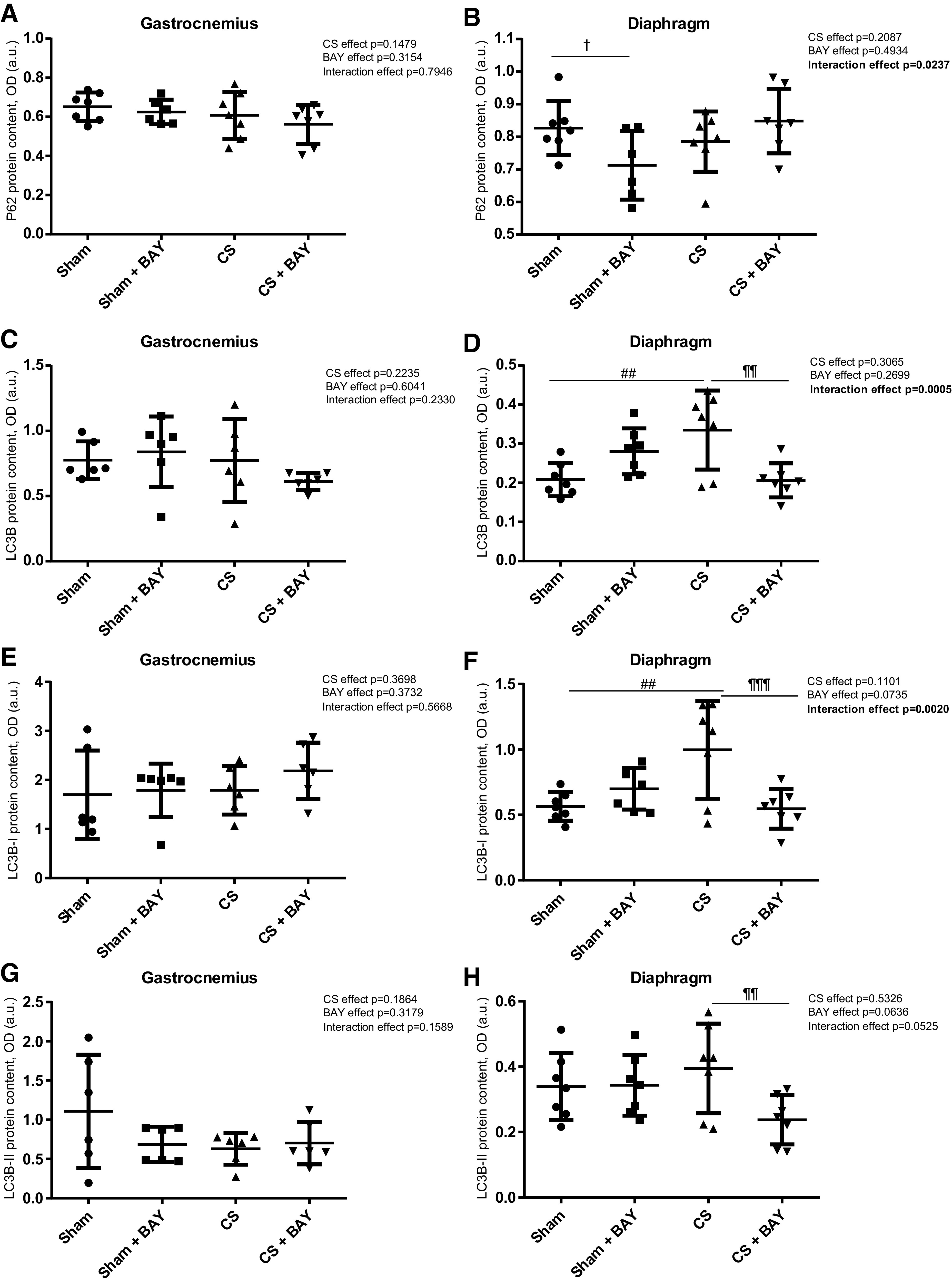
Mean values and standard deviation of the p62 protein content in the gastrocnemius (*A*), p62 protein content in the diaphragm (*B*), LC3B protein content in the gastrocnemius (*C*) and LC3B protein content in the diaphragm (*D*) and isoforms of LC3B-I and II in the gastrocnemius (*E* and *G*) and diaphragm (*F* and *H*) measured as optical density (OD) using arbitrary units (a.u.) in the muscles of the different guinea pig study groups. Potential differences between two groups were analyzed using contrast of marginal linear predictions. Statistical significance: ##*P* ≤ 0.01 between the CS group and the sham control group; ¶¶*P* ≤ 0.01, ¶¶¶*P* ≤ 0.001 between the CS + BAY group and the CS group; †*P* ≤ 0.05 between the sham + BAY group and the sham control group. The effect of CS and treatment and interaction effects are also indicated as actual *P* values for each variable. Bay, Bay 41–2272; CS, cigarette smoke.

**Figure 7.**
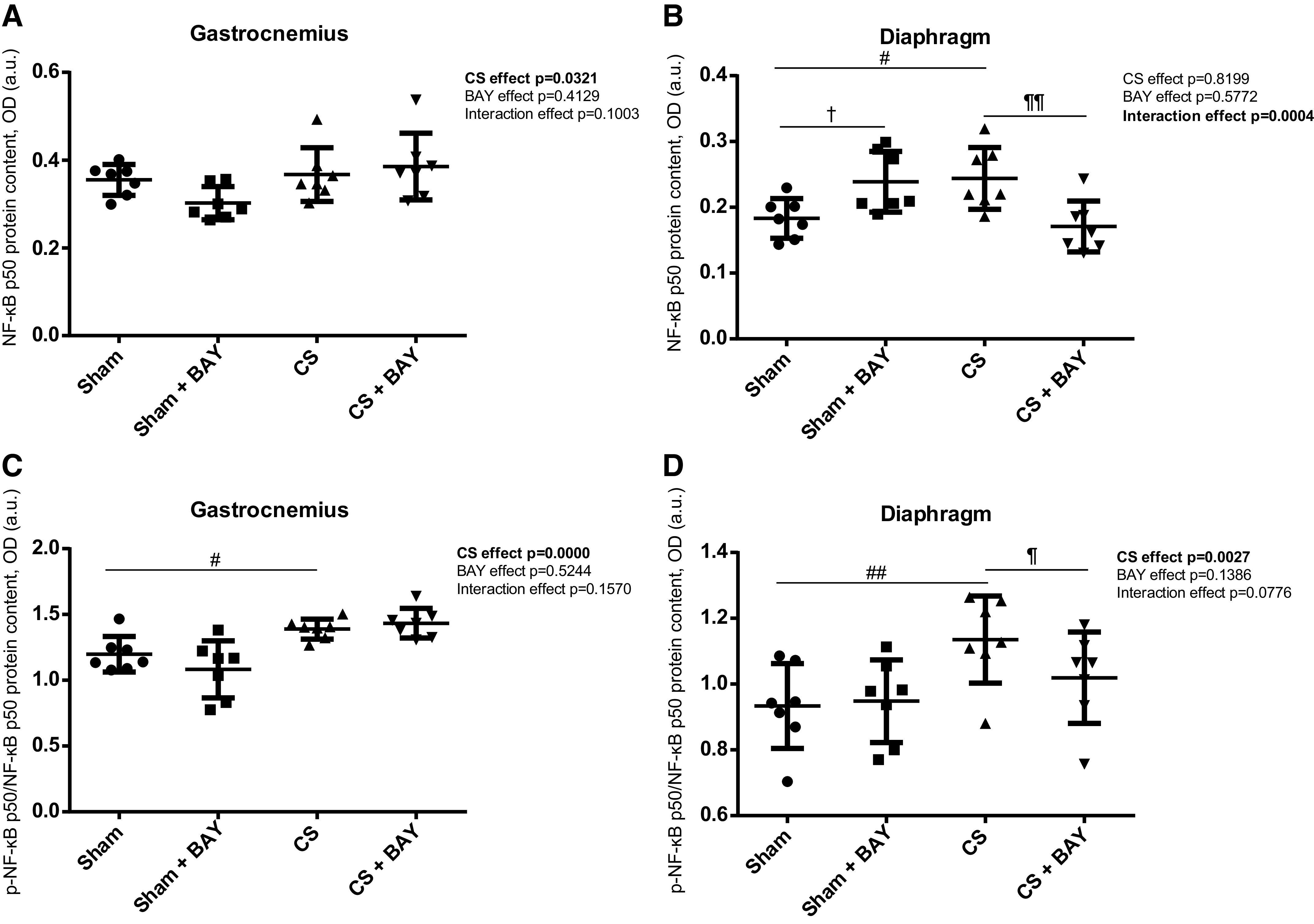
Mean values and standard deviation of the NF-κB p50 protein content in the gastrocnemius (*A*), NF-κB p50 protein content in the diaphragm (*B*), phosphorylated NF-κB p50 protein content in the gastrocnemius (*C*), and phosphorylated NF-κB p50 protein content in the diaphragm (*D*) measured as optical density (OD) using arbitrary units (a.u.) in the muscles of the different guinea pig study groups. Potential differences between two groups were analyzed using contrast of marginal linear predictions. Statistical significance: #*P* ≤ 0.05, ##*P* ≤ 0.01 between the CS group and the sham control group; ¶*P* ≤ 0.05, ¶¶*P* ≤ 0.01 between the CS + BAY group and the CS group; †*P* ≤ 0.05 between the sham + BAY group and the sham control group. The effect of CS and treatment and interaction effects are also indicated as actual *P* values for each variable. Bay, Bay 41–2272; CS, cigarette smoke.

**Figure 8.**
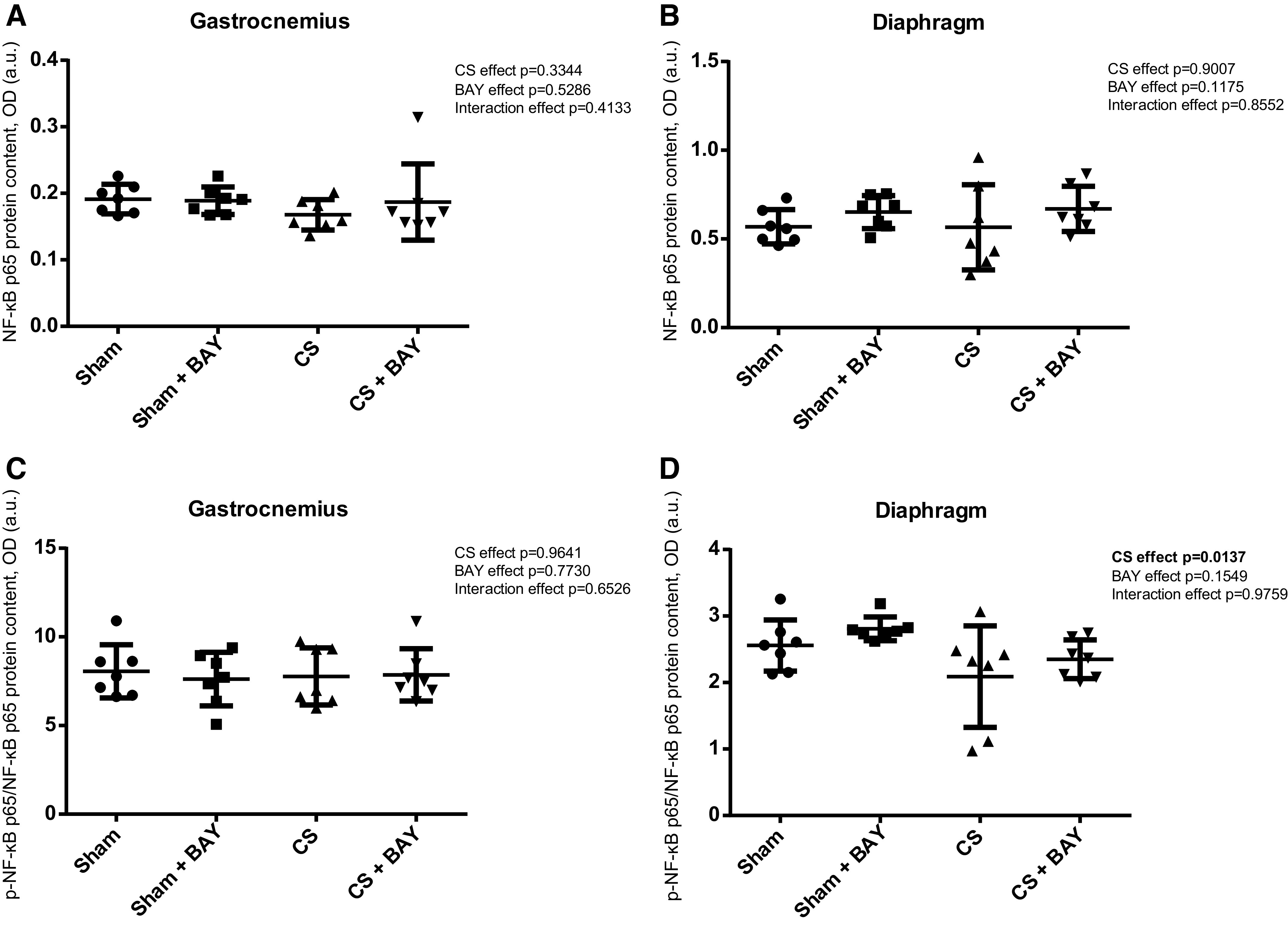
Mean values and standard deviation of the NF-κB p65 protein content in the gastrocnemius (*A*), NF-κB p65 protein content in the diaphragm (*B*), phosphorylated NF-κB p65 protein content in the gastrocnemius (*C*) and phosphorylated NF-κB p65 protein content in the diaphragm (*D*) measured as optical density (OD) using arbitrary units (a.u.) in the muscles of the different guinea pig study groups. The effect of CS and treatment and interaction effects are also indicated as actual *P* values for each variable. Potential differences between two groups were analyzed using contrast of marginal linear predictions. No differences were found between groups.

**Figure 9.**
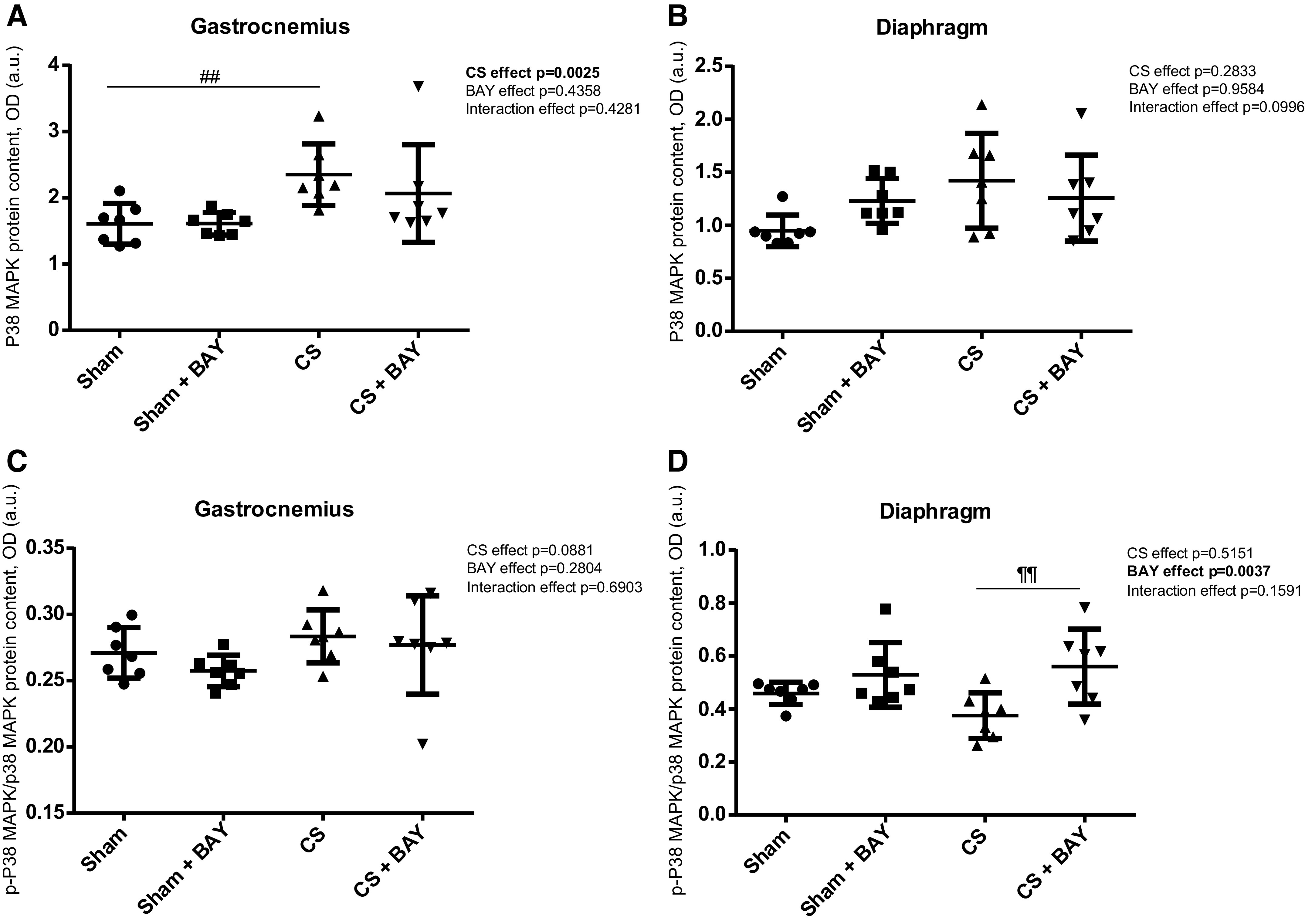
Mean values and standard deviation of the p38 MAPK protein content in the gastrocnemius (*A*), p38 MAPK protein content in the diaphragm (*B*), phosphorylated p38 MAPK protein content in the gastrocnemius (*C*) and phosphorylated p38 MAPK protein content in the diaphragm (*D*) measured as optical density (OD) using arbitrary units (a.u.) in the muscles of the different guinea pig study groups. Potential differences between two groups were analyzed using contrast of marginal linear predictions. Statistical significance: ##*P* ≤ 0.01 between the CS group and the sham control group; ¶¶*P* ≤ 0.01 between the CS + BAY group and the CS group. The effect of CS and treatment and interaction effects are also indicated as actual *P* values for each variable. Bay, Bay 41–2272; CS, cigarette smoke.

Statistical analyses were performed using STATA (software for Statistics and Data Science) software (StataCorp LLC, College Station, TX). All results are expressed as mean values (standard deviation). The normality of the study variables was checked using the Shapiro-Wilk test. The results of the physiological parameters (body weight week 13 and BMI) are represented in Table 1. The results of the structural characteristics muscle fiber type, muscle fiber size, and muscle structural abnormalities are represented in Table 2. The biological variables are represented in Figs. 1–9. Two-way analysis of variance (ANOVA) was used to analyze the following effects: cigarette smoke, treatment with BAY 41–2272, and the interaction between these factors for all the study variables. Moreover, potential differences between two groups were analyzed using contrast of marginal linear predictions. Three levels of comparisons were established for all the study variables: *1*) comparisons between sham and cigarette smoke (CS)-exposed animals, *2*) comparisons between the sham group and the sham group + BAY 41–2272 animals, and *3*) comparisons between the CS group and the CS + BAY 41–2272 animal group. *P* ≤ 0.05 was established as the level of significance. Relationships between variables were assessed using the Pearson’s correlation test

**Table 1.** Physiological parameters in all experimental groups of guinea pig

Physiological Parameters	Sham (*n* = 7)	Sham + Bay (*n* = 7)	CS (*n* = 7)	CS + Bay (*n* = 7)	Bay Effect (*P* Value)	CS Effect (*P* Value)	Interaction Effect (*P* Value)
Body weight week 13, g	918 (57)	976 (75)	802 (57)#	873 (85)	0.021	*P* < 0.001	0.815
BMI, kg/m^2^	9.41 (0.53)	9.89 (0.54)	8.42 (0.51)#	8.79 (0.53)	0.009	*P* < 0.001	0.737

Variables are presented as means (SD). Two-way ANOVA. Potential differences between two groups were analyzed using contrast of marginal linear predictions. “*n*” represents the number of animals studied for each group. Statistical significance is represented as follows: #*P* ≤ 0.05 between CS group and sham control group. The effects of cigarette smoke, Bay 41–2272 treatment, and interaction effect are also indicated as actual *P* values for each variable. Bay, Bay 41–2272; BMI, body mass index; CS, cigarette smoke.

**Table 2.** cGMP levels and structural characteristics of the gastrocnemius and diaphragm muscles in guinea pig study groups

cGMP Levels and Structural Characteristics	Sham (*n* = 7)	Sham + Bay (*n* = 7)	CS (*n* = 6)	CS + Bay (*n* = 7)	Bay Effect (*P* Value)	CS Effect (*P* Value)	Interaction Effect (*P* Value)
*Gastrocnemius muscle*							
cGMP, pmol/mg prot	1.89 (1.18)	2.60 (1.76)	1.10 (0.46)	3.61 (1.51)	0.0050	0.834	0.098
Muscle fiber type, %							
Type I	12.8 (4.4)	9.5 (4.5)	10.4 (7.4)	10.0 (3.6)	0.3496	0.6234	0.4820
Type II	87.2 (4.4)	90.5 (4.5)	89.6 (7.4)	90.0 (3.6)	0.3496	0.6234	0.4820
Muscle fiber size, CSA							
Type I	1,033.7 (238.1)	1,048.7 (311.4)	810.4 (185.8)	791.0 (188.4)	0.9826	0.0225	0.8611
Type II	1,305.8 (255.2)	1,313.2 (244.4)	1,002.2 (259.9)##	1,033.2 (177.6)	0.8381	0.0048	0.8998
Muscle structural abnormalities, %							
Total abnormal fraction	0.37 (0.28)	0.95 (0.54)	1.05 (0.26)#	1.35 (0.65)	0.0240	0.0072	0.4257
Internal nuclei counts	0.10 (0.06)	0.19 (0.17)	0.18 (0.15)	0.30 (0.29)	0.1638	0.2213	0.8658
Inflammatory cell counts	0.08 (0.08)	0.09 (0.11)	0.23 (0.18)	0.19 (0.14)	0.8361	0.0225	0.6496
*Diaphragm muscle*							
cGMP, pmol/mg prot	1.40 (0.48)	3.02 (2.35)	1.15 (0.38)	2.26 (0.68)	0.0090	0.5980	0.3010
Muscle fiber type, %							
Type I	36.7 (5.3)	38.3 (6.9)	32.8 (3.8)	34.2 (5.5)	0.4876	0.0728	0.9687
Type II	63.3 (5.3)	61.7 (6.9)	67.2 (3.8)	65.8 (5.5)	0.4878	0.0727	0.9684
Muscle fiber size, CSA							
Type I	606.7 (96.1)	640.8 (85.2)	538.1 (122.7)	555.4 (63.9)	0.4865	0.0454	0.8192
Type II	915.1 (179.4)	859.4 (121.1)	718.9 (188.6)#	759.1 (177.1)	0.9055	0.0318	0.4676
Muscle structural abnormalities, %							
Total abnormal fraction	0.91 (0.55)	1.29 (0.61)	1.77 (0.45)#	1.35 (0.47)	0.9096	0.0304	0.0537
Internal nuclei counts	0.37 (0.22)	0.66 (0.50)	0.68 (0.41)	0.68 (0.48)	0.3681	0.2952	0.3681
Inflammatory cell counts	0.05 (0.03)	0.12 (0.07)	0.15 (0.14)	0.16 (0.16)	0.4125	0.0961	0.5691

Variables are presented as means (SD). Two-way ANOVA. Potential differences between two groups were analyzed using contrast of marginal linear predictions. “*n*” represents the number of animals studied for each group. Statistical significance is represented as follows: #*P* ≤ 0.05, ##*P* ≤ 0.01 between CS group and sham control group. The effects of cigarette smoke, Bay 41–2272 treatment, and interaction effect are also indicated as actual *P* values for each variable. Bay, Bay 41–2272; CS, cigarette smoke; CSA, cross-sectional area.

## RESULTS

### Physiological Characteristics

#### Effects of CS with respect to controls.

Body weight at *week 13* and BMI significantly decreased (*P* < 0.05 both) in the CS group compared with sham animals (Table 1).

#### Effects of Bay treatment in CS-exposed animals.

A significant increase in body weight and BMI was observed at week 13 in the CS group treated with BAY (Table 1), being very close to the values of the control group.

#### Effects of Bay treatment in nonexposed rodents.

No significant differences were observed in the variables: body weight at *week 13* and BMI between the sham group and the sham group treated with BAY (Table 1).

### cGMP Levels

#### Effects of CS with respect to controls.

Compared with sham-treated animals, in the gastrocnemius of the CS animals, no significant differences were observed in the levels of cGMP. Compared with sham-treated animals, in the diaphragm of the CS animals, no significant differences were observed in the levels of cGMP (Table 2).

#### Effects of Bay treatment in CS-exposed animals.

A significant increase in cGMP levels was observed in gastrocnemius and diaphragm muscles compared with controls.

#### Effects of Bay treatment in nonexposed rodents.

There was a trend toward increased cGMP levels in the gastrocnemius and diaphragm muscles compared with controls, although statistical significance was not reached.

### Structural Characteristics in Limb and Respiratory Muscles

#### Effects of CS with respect to controls.

Compared with sham-treated animals, in the gastrocnemius of the CS animals, no significant differences were observed in the proportions of either slow- or fast-twitch fiber types (Table 2 and Fig. 1). Cross-sectional area (CSA) of slow-twitch fibers did not significantly differ between the two groups, but CSA of fast-twitch fibers was significantly decreased (*P* = 0.0376, Table 2 and Fig. 1). The total abnormal fraction was significantly greater (*P* = 0.0106) in the CS group compared with the sham-treated group (Table 2) and no changes in internal nuclei and inflammatory cell counts were observed (Table 2). Compared with sham-treated animals, in the diaphragm of the CS animals, no significant differences were observed in the proportions of either slow- or fast-twitch fiber types (Table 2 and Fig. 1). Moreover, CSA of slow-twitch fibers did not significantly differ between the two groups, but CSA of fast-twitch fibers was significantly decreased (*P* = 0.0472, Table 2 and Fig. 1). The total abnormal fraction was significantly greater (*P* = 0.0054) in the CS group compared with the sham-treated group (Table 2) and no changes in internal nuclei and inflammatory cell counts were observed (Table 2).

#### Effects of Bay treatment in CS-exposed animals.

No significant differences were detected in the proportions or size of the slow- and fast-twitch fibers or in the proportions of muscle structural abnormalities in the gastrocnemius and diaphragm muscles between CS and CS-treated animals (Table 2).

#### Effects of Bay treatment in nonexposed rodents.

No significant differences were detected in the proportions or size of the slow- and fast-twitch fibers or in the proportions of muscle structural abnormalities in the gastrocnemius and diaphragm muscles between sham and sham-treated animals (Table 2).

### Association between Body Weight and 8-OHdG with Structural Characteristics of Limb and Respiratory Muscles

Data for plasma 8-OHdG levels in the different groups have been previously reported (PlosOne). Plasma levels of 8-OHdG were positively associated with the CSA of the slow-twitch fiber of the gastrocnemius and diaphragm and of the fast-twitch fiber of the diaphragm (Table 3). In addition, a positive correlation was observed between body weight and diaphragm CSA and a trend with gastrocnemius CSA.

**Table 3.** Pearson’s correlation between CSA values and plasma biomarkers

	Gastrocnemius		Diaphragm	
	CSA Type I	CSA Type II	CSA Type I	CSA Type II
Weight	*R* = 0.386	R = 0.249	*R* = 0.654	*R* = 0.552
	*P* = 0.063	*P* = 0.2200	***P* = 0.0002**	***P* = 0.0023**
8-OHdG	*R* = −0.518	R = −0.246	*R* = −0.645	*R* = −0.514
	***P* = 0.00947**	*P* = 0.2260	***P* = 0.0003**	***P* = 0.0061**

CSA, cross-sectional area. Values in bold indicate *P* < 0.05.

### Expression of Proteolytic Markers in Limb and Respiratory Muscles

#### Effects of CS with respect to controls.

In the gastrocnemius, there was an increase in the proteolytic markers MURF1, Atrogin-1, 20 s proteasome subunit C8, and total ubiquitinated proteins (*P* = 0.0351, *P* = 0.0006, *P* = 0.0001 and *P* = 0.0039, respectively) in the CS group when compared with the sham-treated group (Figs. 2, *A* and *C* and 3, *A* and *C*). In the diaphragm of the CS animals, no changes were observed in the proteolytic markers MURF1, Atrogin-1, 20 s proteasome subunit C8, and total ubiquitinated proteins in the CS group when compared with the sham-treated group (Figs. 2, *B* and *D* and 3, *B* and *D*).

#### Effects of Bay treatment in CS-exposed animals.

In the gastrocnemius, the proteolytic markers MURF1, Atrogin-1, 20 s proteasome subunit C8 and total ubiquitinated proteins were significantly decreased (*P* = 0.0105, *P* = 0.0019, *P* = 0.0100 and *P* = 0.0003, respectively) (Figs. 2, *A* and *C* and 3, *A* and *C*) in the CS-treated animals when compared with the CS group. No significant differences were detected in any of the proteolytic markers in the diaphragm in the CS-treated animals when compared with the CS group (Figs. 2, *B* and *D* and 3, *B* and *D*).

#### Effects of Bay treatment in nonexposed rodents.

No significant differences were detected in any of the proteolytic markers in the gastrocnemius between the sham and the sham-treated animals (Figs. 2, *A* and *C* and 3, *A* and *C*). An increase in 20 s proteasome subunit C8 (*P* = 0.0383) was detected in the diaphragm (Fig. 3*B*) in the sham with BAY group when compared with the sham group.

### Expression of Apoptotic Markers in Limb and Respiratory Muscles

#### Effects of CS with respect to controls.

In the gastrocnemius, Bcl-2 and caspase-3 were significantly increased (*P* = 0.0352 and *P* = 0.0002, respectively) (Figs. 4*C* and 5*A*), but no significant changes were detected in Bax protein content and in the number of TUNEL-positive nuclei (Figs. 4*A* and 5*C*) in the CS-exposed group compared with the sham-exposed group. In the diaphragm, the apoptotic markers caspase-3 protein content and the number of TUNEL-positive nuclei were significantly increased (*P* = 0.0143 and *P* = 0.0004, respectively) (Fig. 5, *B* and *D*), whereas Bcl-2 was decreased (*P* = 0.0152) (Fig. 4*D*). No changes were observed in Bax (Fig. 4*B*) in the CS-exposed group compared with the sham-exposed group.

#### Effects of Bay treatment in CS-exposed animals.

No significant differences were detected in any of the apoptotic markers, either in the gastrocnemius or in the diaphragm muscles, in the CS-treated animals when compared with the CS group (Figs. 4 and 5).

#### Effects of Bay treatment in nonexposed rodents.

No significant differences were detected in any of the apoptotic markers in the gastrocnemius when we compared the sham and the sham-treated animals (Figs. 4, *A* and *C* and 5, *A* and *C*). A significant decrease (*P* = 0.0232) in the apoptotic marker Bcl-2 was detected in the diaphragm (Fig. 4*D*) in the sham with BAY group when compared with the sham group.

### Expression of Autophagic Markers in Limb and Respiratory Muscles

#### Effects of CS with respect to controls.

No significant differences were detected in any of the autophagic markers in the gastrocnemius when we compared the CS-exposed and the sham-exposed groups (Fig. 6, *A* and *C*). A significant increase (*P* = 0.0015) in the autophagic marker LC3B or in its isoform LC3B-I was detected in the diaphragm (Fig. 6, *D* and *F*) in the CS group when compared with the sham group.

#### Effects of Bay treatment in CS-exposed animals.

No significant differences were detected in any of the autophagic markers in the gastrocnemius when we compared the CS with BAY and the CS groups (Fig. 6, *A*, *C*, *E*, and *G*). A significant decrease (*P* = 0.0013) in the autophagic marker LC3B or in its isoforms LC3B-I and LC3B-II was detected in the diaphragm (Fig. 6, *D*, *F*, and *H*) in the CS with BAY group when compared with the CS group.

#### Effects of Bay treatment in nonexposed rodents.

No significant differences were detected in any of the autophagic markers in the gastrocnemius when the sham with BAY and the sham groups were compared (Fig. 6, *A* and *C*). A significant decrease (*P* = 0.0412) in the autophagic marker p62 was detected in the diaphragm (Fig. 6*B*) in the sham with BAY group when compared with the sham group.

### Expression of Upstream Signaling Markers in Limb and Respiratory Muscles. Expression of NF-κB Pathway Markers

#### Effects of CS with respect to controls.

A significant increase in the p-NF-κB p50/NF-κB p50 ratio (*P* = 0.0210) was detected in the gastrocnemius when we compared the CS-exposed and the sham-exposed groups (Fig. 7*C*). A significant increase in the total NF-κB p50 subunit and in the p-NF-κB p50/NF-κB p50 ratio (*P* = 0.0106 and *P* = 0.0015, respectively) was detected in the diaphragm (Fig. 7, *B* and *D*) in the CS group when compared with the sham group.

#### Effects of Bay treatment in CS-exposed animals.

No significant differences were detected in any of the markers of the NF-κB pathway in the gastrocnemius when the CS with BAY and the CS groups were compared (Figs. 7, *A* and *C* and 8, *A* and *C*). A significant decrease in the total NF-κB p50 subunit and in the p-NF-κB p50/NF-κB p50 ratio (*P* = 0.0028 and *P* = 0.0280, respectively) was detected in the diaphragm (Fig. 7, *B* and *D*) in the CS with BAY group when compared with the CS group.

#### Effects of Bay treatment in nonexposed rodents.

No significant differences were detected in any of the markers of the NF-κB pathway in the gastrocnemius when the sham with BAY and the sham groups were compared (Figs. 7, *A* and *C* and 8, *A* and *C*). A significant increase in the total NF-κB p50 subunit (*P* = 0.0182) was detected in the diaphragm (Fig. 7*B*) in the sham with BAY group when compared with the sham group.

### Expression of MAPK Pathway Markers

#### Effects of CS with respect to controls.

A significant increase in the total p38 MAPK marker (*P* = 0.0069) was detected in the gastrocnemius when we compared the CS-exposed and the sham-exposed groups (Fig. 9*A*). No significant differences were detected in any of the markers of the MAPK pathway in the diaphragm when the CS and the sham groups were compared (Fig. 9, *B* and *D*).

#### Effects of Bay treatment in CS-exposed animals.

No significant differences were detected in any of the markers of the MAPK pathway in the gastrocnemius when we compared the CS with BAY and the CS groups (Fig. 9, *A* and *C*). A significant increase in the p-p38 MAPK/p38 MAPK ratio (*P* = 0.0030) was detected in the diaphragm (Fig. 9*D*) in the CS with BAY group when compared with the CS group.

#### Effects of Bay treatment in nonexposed rodents.

No significant differences were detected in any of the markers of the MAPK pathway, either in the gastrocnemius or in the diaphragm, in the sham-treated animals when compared with the sham group (Fig. 9).

### Expression of FoxO Pathway Markers

#### Effects of CS with respect to controls.

**Figure 10.**
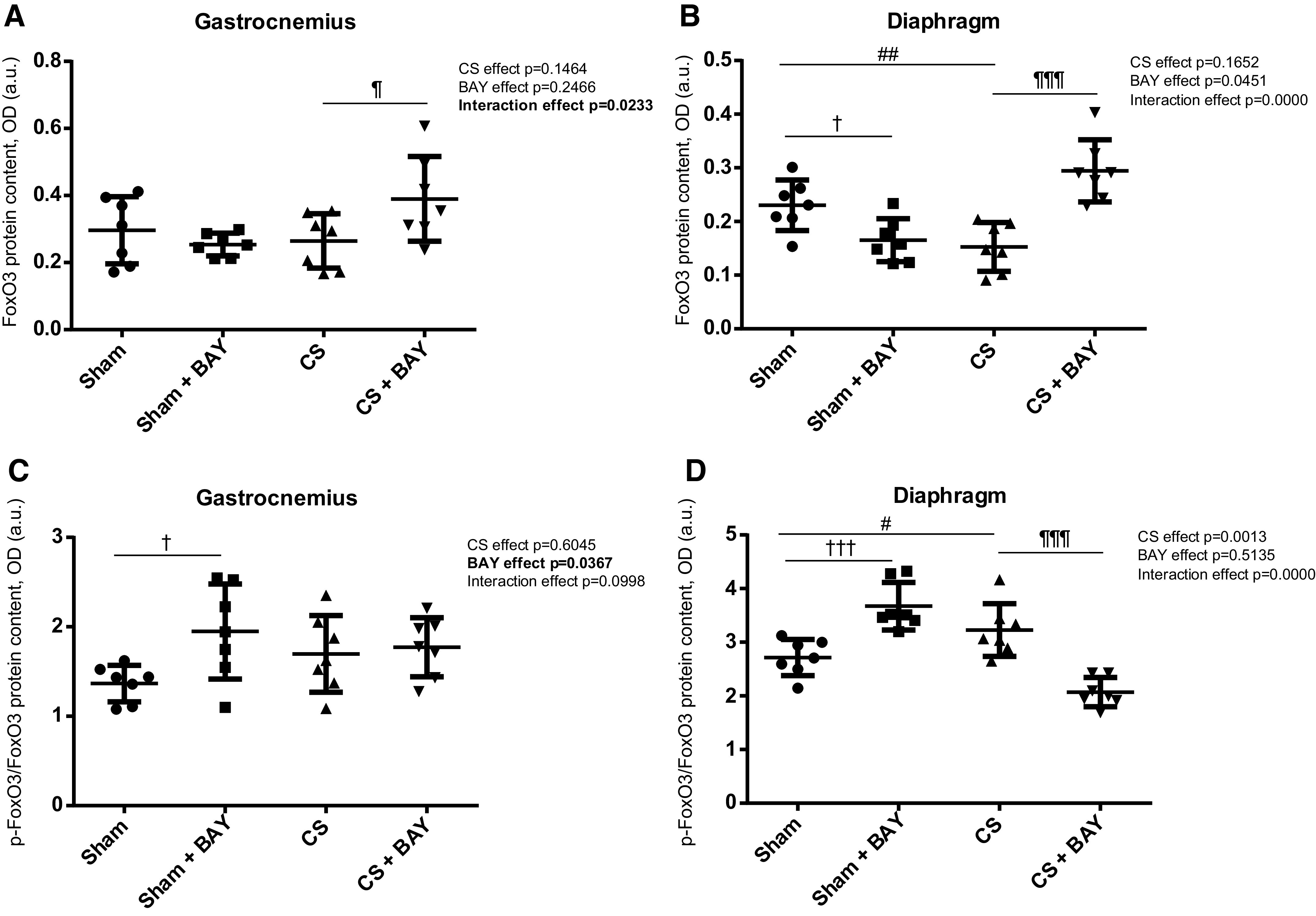
Mean values and standard deviation of the FOXO3 protein content in the gastrocnemius (*A*), FOXO3 protein content in the diaphragm (*B*), phosphorylated FoxO3 protein content in the gastrocnemius (*C*), and phosphorylated FOXO3 protein content in the diaphragm (*D*) measured as optical density (OD) using arbitrary units (a.u.) in the muscles of the different guinea pig study groups. Potential differences between two groups were analyzed using contrast of marginal linear predictions. Statistical significance: #*P* ≤ 0.05, ##*P* ≤ 0.01 between the CS group and the sham control group; ¶*P* ≤ 0.05, ¶¶¶*P* ≤ 0.001 between the CS + BAY group and the CS group; †*P* ≤ 0.05, †††*P* ≤ 0.001 between the sham + BAY group and the sham control group. The effect of CS and treatment and interaction effects are also indicated as actual *P* values for each variable. Bay, Bay 41–2272; CS, cigarette smoke.

No significant differences were detected in FoxO3 marker in the gastrocnemius when we compared the CS-exposed and the sham-exposed groups (Fig. 10, *A* and *C*). A significant increase in the p-FoxO3/FoxO3 ratio (*P* = 0.0239) and a significant decrease in the total FoxO3 marker (*P* = 0.0060) was detected in the diaphragm (Fig. 10, *B* and *D*) in the CS group when compared with the sham group.

#### Effects of Bay treatment in CS-exposed animals.

A significant increase (*P* = 0.0175) in total FoxO3 was detected in the gastrocnemius when we compared the CS with BAY and the CS groups (Fig. 10*A*). A significant decrease in the p-FoxO3/FoxO3 ratio (*P* = 0.0000) was detected in the diaphragm (Fig. 10*D*) in the CS with BAY group when compared with the CS group. A significant increase in total FoxO3 marker (*P* = 0.0030) was detected in the diaphragm (Fig. 10*B*) in the CS with BAY group when compared with the CS group.

#### Effects of Bay treatment in nonexposed rodents.

A significant increase in the p-FoxO3/FoxO3 ratio (*P* = 0.0105) was detected in the gastrocnemius when the sham with BAY and the sham groups were compared (Fig. 10*C*). A significant increase in the p-FoxO3/FoxO3 ratio (*P* = 0.0001) was detected in the diaphragm (Fig. 10*D*) in the sham with BAY group when compared with the sham group. Finally, a significant decrease in the total FoxO3 marker (*P* = 0.0183) was detected in the diaphragm (Fig. 10*B*) in the sham with BAY group when compared with the sham group.

## DISCUSSION

In this study, we evaluated the effects of the sGC stimulator BAY 41–2272 administration in a preclinical model of COPD as a potential treatment for skeletal muscle dysfunction. Chronic exposure to CS led to significant weight loss in guinea pigs. Weight loss was associated with increased levels of proteolytic markers of muscle atrophy MURF-1, Atrogin-1, 20 s proteasome C8 subunit, and total protein ubiquitination mainly in the gastrocnemius, whereas the size of fast-twitch muscle fibers decreased significantly. In CS-exposed animals, long-term treatment with BAY 41–2272 resulted in a significant reduction in the gastrocnemius levels of the aforementioned proteolytic markers, concomitant with weight recovery, with increased cGMP bioavailability, and a decrease in apoptosis values, although the latter did not reach statistical significance. Both limb and respiratory muscles of CS-exposed animals also showed increased activation levels of upstream markers related to muscle atrophy, whereas treatment with BAY 41–2272 significantly attenuated the activation of these markers in the diaphragm. A remarkable point is that levels of some of the biomarkers analyzed differed between respiratory and limb muscles.

The most prominent result of this study was that treatment with BAY 41–2272 was able to normalize in the gastrocnemius of CS-exposed animals the increased levels of specific proteolytic markers of myosin degradation (29), such as MURF1 and Atrogin-1. The mechanisms of skeletal muscle dysfunction leading to muscle atrophy in COPD are known to occur through different nonexclusive cellular processes (30), but the hallmark of muscle atrophy is accelerated intracellular protein degradation (31, 32), which occurs through dysregulation of two major mechanisms, i.e., the ubiquitin-proteasome (17) and the lysosomal (33, 34) pathways. In our study, BAY 41–2272 was able to reverse and normalize the expression levels of the ubiquitin-proteasome pathway, indicating that the beneficial effect of this drug in reversing weight loss may have been through a normalization of the rate of protein degradation. The mechanism underlying long-term treatment could be improved cGMP bioavailability and/or reduced oxidation of sGC, as shown in previous studies (7, 19). However, it is interesting to note that this apparent decrease in proteasome activity following long-term treatment with BAY 41–2272 contrasts with the fact that, in experiments performed in vitro on cell cultures, PKG rapidly increases cytosolic proteasomes, protein ubiquitination and overall protein degradation (35). In that in vitro study, activation of the proteasome by the stimulation of cGMP-PKG axis ensued within a few hours after incubation with the sGC stimulator and would have a physiological action of stimulating the selective proteasomal degradation of misfolded proteins (toxic proteins) (36). However, there are no in vitro studies on long-term treatment and modification of PKG activity, but our results suggest complex mechanisms in long-term regulation that require further analysis.

Remarkably, the increase in proteolytic markers that we observed in the gastrocnemius muscle was not observed in the diaphragm of animals exposed to CS. These differences could be explained due to diaphragm activity versus limb muscle deconditioning (37, 38). In contrast, the autophagy biomarker LC3B only increased in the diaphragm of CS-exposed animals and decreased with BAY 41–2272 treatment, whereas significant changes were not detected in the gastrocnemius. Although LC3B is usually associated with a role in the autophagic pathway, previous studies have described several additional alternative functions, including a protective role in the development of hypoxia-induced pulmonary hypertension (PH) via the HIF-1α pathway (39). In addition, increased cellular ROS production is known to promote HIF-1α stabilization (40), a mechanism that would be upregulated in our CS-exposed animals (7). In this regard, we observed a stronger correlation between the plasma oxidation marker 8-OHdG and diaphragm muscle fiber size. Importantly, the CS-exposed animals developed pulmonary hypertension (20), suggesting that the increase in LC3B in the diaphragm could somehow be associated with this complication. Finally, ROS could also inhibit autophagy causing an increase in the lipidated form of LC3 (41). Further studies regarding the role of LC3B would be necessary to demonstrate alternative function to autophagy in respiratory muscles.

Transcriptional regulation is a key component of autophagy regulation in skeletal muscle (42). A large number of studies have demonstrated that autophagy is under the control of multiple transcription factors such as FOXO and NF-κB (43, 44). FOXO proteins, and more specifically FOXO3, functions as an activator of the transcription of autophagy genes whereas NF-κB orchestrates a broad range of cellular processes, among them, cell division and differentiation and cell death and survival (45). NF-κB signaling has been shown to be involved in autophagy in a context-dependent manner (46). This is done regulated by the phosphorylation of the NF-κB subunit p50 and by the level of heterodimer NF-κB p50-NF-κB p65 (47, 48). We evaluated the expression of these upstream signaling biomarkers in limb and respiratory muscles and observed a significant decrease in the total level of FOXO3 in the diaphragm and a significant increase in the p-NF-κB p50/NF-κB p50 ratio in both the gastrocnemius and diaphragm of CS-exposed animals. Treatment with BAY 41–2272 induced a significant decrease in the total NF-κB p50 subunit and in the p-NF-κB p50/NF-κB p50 ratio and a significant increase in total FOXO3 in the diaphragm, whereas no significant changes were detected in the gastrocnemius. We do not know the exact mechanism by which sGC may reverse transcriptional factors of autophagy, but downstream, activation of the IGF-I/phosphatidylinositol 3-kinase/protein kinase B (AKT) pathway, which is essential for muscle cell survival and hypertrophy, entails the inhibition of MURF1 and Atrogin-1 (49). Once activated, AKT phosphorylates FOXO1 and FOXO3 proteins. They are then excluded from the nucleus and become transcriptionally inactive (49). However, AKT also phosphorylates NF-κB so it becomes a regulatory hub for skeletal muscle cell survival and proliferation. It is noteworthy that, in endothelial cells, eNOS can be activated by AKT-dependent phosphorylation, and elevated eNOS activity is sufficient to activate PI3K/AKT signaling via PKG and induce cell migration and angiogenesis through NO/cGMP/PKG (50). Thus, this important pathway could explain the enhancement of Atrogin-1 and FOXO3 levels in skeletal muscle cells. Therefore, a possible mechanism by which autophagy processes could diminish in skeletal muscle after the stimulation of sGC could be through increased phosphorylation of AKT via PKG.

Muscle wasting in COPD may be also mediated through the regulation of the p38 mitogen-associated protein kinase (MAPK) signaling pathway, since the activation of p38 may be at least partially sufficient for the development of some muscular dystrophy phenotypes (51). Wissing et al. (52) showed that these effects were due to p38 phosphorylating and activating the proapoptotic factor Bax. These data suggest that inhibition of p38 may be beneficial for the treatment of multiple forms of muscular dystrophy by preventing inflammation and myofiber death. Nevertheless, Riddoch-Contreras et al. (53) studied p38 MAPK signaling in skeletal muscle from stable patients with COPD and, despite the presence of reduced fat-free mass and quadriceps weakness, found no upregulation. Analogously, in our study, guinea pigs exposed to CS only showed a significant increase of the total p38 MAPK marker in the gastrocnemius, but treatment with BAY 41–2272 did not significantly reduce its expression level. Only the p-p38 MAPK/p38 MAPK ratio was increased after BAY 41–2272 treatment in the diaphragm, suggesting a potential action on its specific inhibitor dual-specificity phosphatase DUSP1. In line with these results, CS-exposed animals showed discrete increases in apoptotic marker levels, which were not reversed by BAY 41–2272 treatment.

In this study, we consider two limitations to be taken into account. First, we did not record the nocturnal activity of the animals, since a more active or more sedentary behavior due to CS could also be associated with some changes in the different biomarkers of muscle function. Second, the samples were obtained 24 h after the last dose of CS or drug administration, allowing time to recover, at least partially, the preexisting levels of some biomarkers. In addition, the animals received a daily dose of Vit C supplement, in addition to the vehicle containing PEG400, both with antioxidant properties, which could have reduced the differences between groups.

### Conclusions

In this animal model of COPD, exposure to CS led to skeletal muscle abnormalities, which were of greater magnitude within the peripheral muscles than in the main respiratory muscle; increased proteolysis may have largely contributed to these findings. The enzyme sGC, through a cGMP-PKG signaling mechanism, plays an important role in muscle fatigue and fiber type specification and is associated with cigarette smoke exposure, thus suggesting that targeting sGC might exert beneficial effects on muscle alterations in patients with COPD.

## ETHICAL APPROVALS

All procedures were approved by the ethics committee for animal experimentation of the University of Barcelona (registry: 383/15). An initial set of assessments in these animals has been previously published (20).

## DATA AVAILABILITY

Data will be made available on reasonable request.

## GRANTS

The study has been funded by grants from the Instituto de Salud Carlos III (ISCIII) (Projects PI16/01147, PI18/00075, PI18/00960, and Miguel Servet CP17/00114) and cofunded by FEDER “Una manera de hacer Europa,” an unrestricted educational grant from Bayer AG and the Spanish Society of Respiratory Medicine and Sociedad Española de Neumología y Cirugía Torácica (SEPAR 2019; SEPAR 2020).

## DISCLAIMERS

None of the funding agencies played any role in the design of the study and collection, analysis, and interpretation of data and in writing the manuscript.

## DISCLOSURES

No conflicts of interest, financial or otherwise, are declared by the authors.

## AUTHOR CONTRIBUTIONS

V.I.P. and J.A.B. conceived and designed research; M.G. and T.P. performed experiments; M.G. and E.B. analyzed data; V.I.P., I.B., O.T-C., T.P., J.A.B., and E.B. interpreted results of experiments; M.G. prepared figures; V.I.P. and E.B. drafted manuscript; V.I.P., J.A.B., and E.B. edited and revised manuscript; V.I.P., I.B., O.T-C., T.P., J.A.B., and E.B. approved final version of manuscript.
